# Green fabrication of ZnO nanoparticles via *spirulina platensis* and its efficiency against biofilm forming pathogens

**DOI:** 10.1186/s12934-024-02360-x

**Published:** 2024-03-27

**Authors:** Nashwa A. Ahmed, Amal S. Othman

**Affiliations:** 1 Lecturer of Microbiology, Faculty of Applied Health Sciences Technology, October 6 University, Giza Governorate, Egypt; 2 Assistant professor of Microbiology, Faculty of Applied Health Sciences Technology, October 6 University, Giza Governorate, Egypt

**Keywords:** *Spirulina platensis*, *ZnO nanoparticles*, Antimicrobial, Antibiofilm formation, Antioxidant, Anti-inflammatory, Anticoagulant, Antitumor, Cytotoxicity

## Abstract

Excessive consumption of antibiotics is considered one of the top public health threats, this necessitates the development of new compounds that can hamper the spread of infections. A facile green technology for the biosynthesis of Zinc oxide nanoparticles (ZnO NPs) using the methanol extract of *Spirulina platensis* as a reducing and stabilizing agent has been developed. A bunch of spectroscopic and microscopic investigations confirmed the biogenic generation of nano-scaled ZnO with a mean size of 19.103 ± 5.66 nm. The prepared ZnO NPs were scrutinized for their antibacterial and antibiofilm potentiality, the inhibition zone diameters ranged from 12.57 ± 0.006 mm to 17.33 ± 0.006 mm (at 20 µg/mL) for a variety of Gram-positive and Gram-negative pathogens, also significant eradication of the biofilms formed by *Staphylococcus aureus* and *Klebsiella pneumoniae* by 96.7% and 94.8% respectively was detected. The free radical scavenging test showed a promising antioxidant capacity of the biogenic ZnO NPs (IC_50=_78.35 µg/mL). Furthermore, the anti-inflammatory role detected using the HRBCs-MSM technique revealed an efficient stabilization of red blood cells in a concentration-dependent manner. In addition, the biogenic ZnO NPs have significant anticoagulant and antitumor activities as well as minimal cytotoxicity against Vero cells. Thus, this study offered green ZnO NPs that can act as a secure substitute for synthetic antimicrobials and could be applied in numerous biomedical applications.

## Introduction

The emergence and spread of antibiotic-resistant microorganisms endanger human and animal health and result in a global health disaster [1]. Furthermore, the diversity of stimuli and free radical stimulators makes it difficult for biological systems’ antioxidant defenses to effectively combat oxidative stress [2]. Green approaches for the synthesis of new compounds that have antioxidant and antibacterial properties are among the innovative methods for addressing these issues [3]. Nanomaterials which are now known as the miracles of modern medicine have enormous potential in a variety of scientific domains because of their physicochemical and biological properties [4], metal oxide nanoparticles are among the wide range of nanoparticles that are accessible, they are regarded to be the most promising based on their unique characteristics like mobility, excellent biocompatibility, adhesiveness [5], and higher surface to volume ratio if compared with the larger size of the bulk materials they were made of [6–10]. Previous reports highlighted the various applications of metal oxide nanoparticles as follows (Table 1):

**Table 1:** Applications of different types of metal oxide nanoparticles

No	Type of Nanoparticle	Applications	References
1	TiO_2_ nanoparticles	Antibacterial, Photodegradation, Removal of heavy metals	7
2	Ag_2_O nanoparticles	Antimicrobial, Anti-inflammatory, Biomedical applications	8
3	CuO nanoparticles	Antibacterial, Antioxidant, Photocatalytic degradation, Biomedical applications	9
4	FeO nanoparticles	Wastewater treatment, Photodegradation	10
5	ZnO nanoparticles	Antimicrobial, Antioxidant, Anti-inflammatory, Dermato-cosmetics, Wastewater treatment	11

Zinc oxide nanoparticles (ZnO NPs) recently caught the interest of numerous scientists due to their unique distinct features and advantageous applications in various industrial and biomedical scopes including medicine administration, photocatalytic degradation, paints, electrical devices, cosmetics, and personal care items [11]. ZnO NPs exhibit diverse activities against multidrug-resistant bacteria, also their combination with antibiotics can help to reduce microbial resistance due to the variation of their mode of action [12]. Based on previous literature ZnO NPs were synthesized via physical or chemical routes, physical methods require high energy consequently high cost and produce low yield of nanoparticles [13], also chemical methods are dangerous due to the involvement of hazardous chemicals [14], so there is a growing need to develop an ecofriendly, sustainable green methodology for the synthesis of nanoparticles [15], Biological methods of nanoparticle synthesis using microorganisms, enzymes, and plant or plant extracts [16] have been suggested as possible ecofriendly alternatives to chemical and physical methods, Klaus et al. [16] reported that both unicellular and multicellular microorganisms are known to produce nanoscaled inorganic materials either intracellularly or extracellularly, Cyanobacteria were reported to have a termendous role in the green production of biomineral structures and metal nanoparticles [17], they have an advantage over terrestrial plants as they do not compete for agricultural land or freshwater resources also they can be obtained in huge biomass at low cost [18]. *Spirulina platensis* which is a nutritious free-floating filamentous photoautotrophic blue-green micoalgae is an important representative of these microorganisms. It is characterized by cylindrical, multicellular trichomes in an open, left-hand helix, it can be found in great biomass in soils, brackish water, rivers, and ponds [19], it includes a high quantity of protein along with all the required amino acids and other important nutrients, its therapeutic use has drawn more interest from researchers in a variety of fields [20], also it is applied in wastewater treatment especially confectionary waste effluents [21], furthermore the microalgal extract has shown enormous potential for use in many biotechnological fields as they are wealthy with various bioactive molecules that were authenticated as potential reducing/stabilizing agents in metal nanoparticles formation [22]. ZnO NPs have excellent interaction with microorganisms, due to their high ratio of surface area to volume and minimized size, their bactericidal mechanism of action depends on several parameters, such as their morphology, composition, and concentration [23] Several studies have reported that ZnO NPs possess selective toxicity to both Gram-positive and Gram-negative bacteria such as *Escherichia coli*, *Salmonella* sp. and *Staphylococcus aureus* but displayed minimal effect on human cells [24]. Another previous report revealed the bioefficiency of ZnO NPs synthesized using plantain peel extract against *Staphylococcus aureus* 26,923, *Bacillus cereus* MTCC 430, *Salmonella enterica*, and *Klebsiella pneumoniae* with minimum inhibitory concentration (MIC) of 100 µg/mL against the tested strains [25]. Biofilm creation is a public health issue as it is the major cause of multidrug resistance Khan et al. [26] reported that ZnO NPs suppress the growth and biofilm formation of a wide range of bacterial isolates. Therefore, it was of considerable importance to scrutinize the role of ZnO NPs underlining their antimicrobial toxicity against well-known human pathogens including both Gram-negative and Gram-positive pathogenic bacterial isolates. According to the previous regards, this study aimed to report a novel eco-friendly sustainable source for large-scale production of green ZnO NPs using *S. platensis* methanol extract and ensuring its characterization using microscopic and spectroscopic routs, in addition, the evaluation of the antimicrobial and anti-biofilm formation of the green synthesized ZnO NPs against Gram negative and Gram positive bacteria, also evaluation of the potentiality of the antioxidant, anti-inflammatory, antitumor and anticoagulant actions as well as cytotoxicity of the the biogenic ZnO NPs.

## Materials and methods

### Preparation of the algal extract

Fresh algal material of *Spirulina platensis* (*S. platensis*) was kindly obtained from the Biotechnology Unit, National Research Center (NRC), Egypt. Algal samples were washed with distilled water many times to remove any impurities and then freeze-dried, double extraction procedure was completed according to Martelli et al. [17], 10 g of the pulverized *S. platensis* were mixed with 100 mL of methanol/water (70:30 v/v) and allowed to stand for 24 h with shaking in dark conditions, the sample was centrifuged (Eppendorf 5800 Centrifuge, Model 5810R, Hamburg, Germany) to recover the solid components which were subjected to the next extraction step, the combined filtrates were concentrated and dried under vacuum using Strike 300 rotary evaporator (Steroglass, Perugia, Italy). The dried powder was kept for further experiments.

### Qualitative chemical screening of the methanol extract

*S. platensis* extract was injected in a Trace GC-TSQ Quantum mass spectrometer (Thermo Scientific, Austin, Texas, USA) to analyze the extract contents using a direct capillary column TG-5MS (30 m x 0.25 mm x 0.25 m film thickness). The column oven’s temperature was originally maintained at 50 °C before being raised by 5 °C/min to 200 °C and maintained for 2 min. The temperature was upped to 290 °C, then it was held for two minutes. Temperatures were maintained at 270 °C and 260 °C for the MS transfer line and injection respectively. Helium was used as the gas phase, flowing at a steady rate of 1 mL/min [27].

### Green synthesis of ZnO nanoparticles (NPs)

**Fig. 1:**
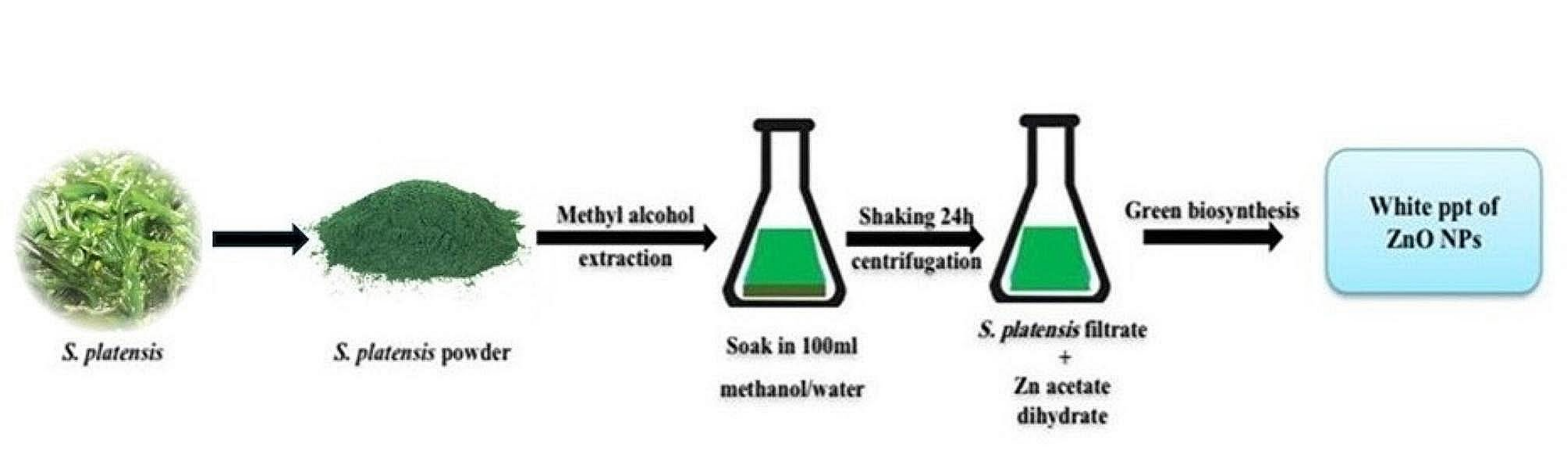
Schematic illustration of the sustainable green synthesis of ZnO NPs

Fifty milliliters of 10 mM Zn (CH_3_COO)_2_.2H2O (Sigma–Aldrich, St. Louis, MO, USA) solution was stirred via a magnetic stirrer for 30 min, 10 mL of *S. platensis* freshly prepared methanol extract (1% w/v) was added dropwise, and stirring was done at 800 rpm for 3–4 h at 50 °C till the formation of a cream-colored zinc hydroxide precipitate. The solution mixture was allowed to sit for 30 min to reduce the zinc hydroxide. centrifugation of the reaction mixture (at 4100 rpm for 12 min) was conducted; the whitish sediments were collected and washed with distilled water. Pure ZnO NPs were obtained by calcinating the ZnO NPs mixture for 4 h at 455 °C in a muffle furnace (Fig. 1) [28].

### Characterization of the green synthesized ZnO-NPs

#### UV-visible scan

For determination of the optical characters of the biogenic ZnONPs 1mL of 96% ethanol was used to disseminate 0.01 g of prepared nanoparticles. Using a UV-visible spectrophotometer (Genway, Australia), the spectra were captured during a 200–800 nm wavelength scan, *S. platensis* methanol extract was used as blank.

#### Field emission-scanning electron microscopy (FE-SEM) and transmission electron microscopy (TEM)

FE-SEM (Sigma, Germany) equipped with an energy-dispersive X-ray spectrometer (EDX, Bruker, Germany) was used to analyze the topography and morphology of the biologically synthesized nanoparticles to determine their chemical compositions. The film on the FE-SEM grid was then dehydrated by exposing it to gold for 5 min after a part of the sample was placed on a carbon-coated copper (CCC) grid. The shape and size distribution of the bioproduced ZnO NPs were confirmed by using TEM (JEM-2100, JEOL, Tokyo, Japan) at an accelerated voltage of 200 kV.

#### Fourier transform infra-red spectroscopy (FTIR) analysis

FTIR analysis (PerkinElmer, Germany) was used to determine the functional groups in the biologically synthesized nanoparticles. The spectra were examined with a frequency of 4.0 cm^− 1^ at a wavelength of 4000 − 400 cm^− 1^.

#### X-ray diffraction (XRD)

The crystalline structure of the green synthesized ZnO NPs was determined by an X-ray diffractometer (PerkinElmer, Germany) with Cu-Kα radiation (λ = 1.5406 Angstrom). Relative intensity measurements were made throughout a 2θ range of 5°–80°, 2θ readings and relative proportions (I/Io) were calculated from the chart, JCPDS charts were used to identify the elements in the metal components.

### Antibacterial vulnerability

Standard bacterial strains represented by *Staphylococcus aureus* (*S. aureus*) ATCC 25,923, *Streptococcus pyogenes* (*S. pyogenes*) ATCC 19,561, *Salmonella typhi* (*S. typhi*) ATCC 14,028 and *Klebsiella pneumonia* (*K. pneumonia*) ATCC 13,883, were kindly obtained from the Faculty of Pharmacy, Cairo University, Egypt, and maintained in their specific media at 30 ºC for 24 h, then kept at 4 ºC. The Agar well diffusion assay was utilized to ascertain the biogenic nanoparticles’ antibacterial efficacy against the examined pathogens [29]. Firstly, fresh cultures were used to create bacterial suspension using 0.85% sterilized saline solution, the turbidity was adjusted at 0.5 McFarland standard (10^8^ CFU/mL), 0.5 mL of each bacterial suspension was spread homogeneously on fresh Mueller–Hinton agar plates. Using a sterile borer, wells (6 mm) were created, and 100 µL of (10–20 µg/mL in DMSO) biosynthesized ZnO NPs were loaded, the inoculated plates were kept at room temperature for 2 h and then incubated at 37 °C for 24 h, the width of each inhibitory zone (mm) was detected, DMSO and tetracycline (25 µg/mL) were used as negative and positive controls respectively.

### Estimation of MIC and MBC

The minimum inhibitory concentration (MIC) was ascertained against the tested pathogenic strains via the micro-broth dilution method, this assay was conducted by performing two-fold serial dilutions of ZnO NPs spanning from 500 to 3.91 µg/mL. The bacterial cultures were incubated for 24 h at 37ºC, and the growth was monitored spectrophotometrically at 600 nm [30]. To match the results, DMSO and tetracycline (25 µg/mL) were used as negative and positive controls respectively, for estimating the bactericidal effect (MBC), 10 µL of culture from the wells with no visible bacterial growth was taken, distributed on plates containing nutrient and further incubated for 12 h at 37ºC, the lowest concentration of the ZnO NPs where the bacterial growth was not detected, was taken as the minimum bactericidal concentration (MBC) for the examined strains.

### Estimation of morphological distortions of bacterial cells through transmission electron microscope examination (TEM)

To examine ultrastructure variation after being treated with green ZnO NPs, the inocula of the most sensitive bacterial pathogens were fixed with 2.5% glutaraldehyde for two hours. Following a two-hour treatment with 2% osmium tetroxide, the blocks were dyed with 1% uranyl acetate and then dried with a graduated ethanol series. After that, resin was used to insert the specimens. The materials were divided into slices using an ultra-microtome (Leica, Wetzkar, Germany) and the sections were examined under the TEM (JEM-2100, JEOL, Tokyo, Japan) [31].

### Microtiter plate assay for biofilm quantification

The quantitative effect of green ZnO NPs on biofilm formation was estimated by microtiter plate assay as performed by Stepanovic et al. [32]. A volume of 20 µL of each suspension (0.5 McFarland) of the tested bacterial isolates (the performance of biofilm formation was done in an earlier study) was aliquoted in the wells of a sterile 96-well polystyrene microtiter plate followed by 180 µL of tryptone soya broth (TSB, OXOID), after 18 h of incubation at 37ºC the plates were rinsed with sterile phosphate buffer to remove free cells, the remaining attached bacteria were resuspended with 100 µL of Muller Hinton broth (MHB, OXOID) and challenged with 100 µL of varying concentrations (0.5MIC, 1MIC, 2MIC) of ZnO NPs, in positive control wells no treatment with ZnO NPs was given, while in negative control wells (blank) 200 µL of MHB only were added. The microtiter plates were kept at 37ºC in a static incubator, after 24 h the polystyrene plates were emptied and then gently washed three times with sterile phosphate buffer to remove free-floating cells, wells were then stained with crystal violet solution (0.1%) for 20 min. the excess amount of crystal violet was washed gently with sterile phosphate buffer and allowed to air-dry for 20 min at room temperature. Ultimately, 250 µL of 95% ethanol was added to each well to solubilize the dye that was attached to the cells. The absorbance was then measured at 620 nm using a microplate reader after 15 min of incubation. All experiments were performed in triplicates for each isolate, mean values and standard deviations were calculated the percentage inhibition of biofilm formation according to the following equation [33]:

Biofilm inhibition percentage = 100 - [(OD sample / OD control) x 100]

### Biological evaluation of ZnO NPs

#### Antioxidant activity

Green ZnO NPs capacity to neutralize free radicals was assessed using the 1,1-diphenyl-2-picrylhydrazyl (DPPH) method, 0.1 mM DPPH solution in ethanol was prepared, the reagent bottle was covered with aluminum foil, and stand for 1 h in dark. ZnO NPs samples were prepared at different concentrations (3.9, 7.8, 15.62, 31.25, 62.5, 125, 250, 500, 1000 µg/mL), then 3 mL of each ZnO NPs sample was mixed with 1 mL of DPPH solution. The tubes were vigorously shaken before being left to stand at room temperature for 30 min, a noticeable color shift from purple/violet to yellow was observed as a result of the scavenging activity. UV spectrophotometer (UV-VIS Milton Roy, Australia) was used to measure the absorbance of samples at 517 nm, the antioxidant activity of the nanoparticles was compared with that of the control. All experiments were performed in triplicates [34].

#### Anti-inflammatory activity

To evaluate in vitro anti-inflammatory efficacy of prepared ZnO NPs, the human red blood cells (HRBCs)-membrane stabilization technique (HRBCs-MSM) has been used according to the outlined procedure by Anosike et al. [35].

#### Anticoagulation activity

The anticoagulant activity of the green-prepared ZnO NPs was investigated by the classical anticoagulant assays for PT and PTT [36].

#### Antitumor activity and cytotoxicity assay

To determine the antitumor activity of green ZnO NPs, Caco-2 cell lines were purchased from VACSERA, under standard conditions the cells were cultured in Dulbecco’s modified Eagle’s medium, then kept at 37 °C in a humidified environment with 5% CO_2_. Normally untreated and treated cells with nanoparticles were done. The medium was then aspirated and stained with crystal violet. Glacial acetic acid (30%) was then added to each well after the stain was removed, the absorbance of the plates was then recorded at 490 nm [37]. Vero cells were employed to evaluate the ZnO NPs’ safety, at a density of 6 × 10^4^ cells /well, the cells were floated in 96-well tissue culture plates with 100 µL of liquid in each well before being incubated for 24 h. The cells in the plates were then treated with 10 µL of ZnO NPs diluted in 0.5% DMSO at doses of 1000, 500, 250, 125, 62.5, 31,25, 15.6,7.8, 3.9, 2, and 0 µg/mL, cells were cultured with and without nanoparticles on the plates, after 24 h of incubation in CO_2_ incubator, crystal violet was added, followed by a distilled water wash and 30% glacial acetic acid. Viable cells were then detected at 490 nm [38].

### Statistical analysis

Data was analyzed using the statistical package for social sciences, version 23.0 (SPSS Inc., Chicago, Illinois, USA). The quantitative data were presented as mean ± standard deviation and ranges, also qualitative variables were presented as numbers and percentages. A one-way analysis of variance (ANOVA) when comparing more than two means. Post Hoc test: Tukey’s test was used for multiple comparisons between different variables. The confidence interval was set at 95% and the margin of error accepted was set at 5%. P-value < 0.05 was considered significant, P-value < 0.001 was considered as highly significant, and P-value > 0.05 was considered insignificant.

## Results and discussion

### Chemical screening of the algal extract

**Fig. 2:**
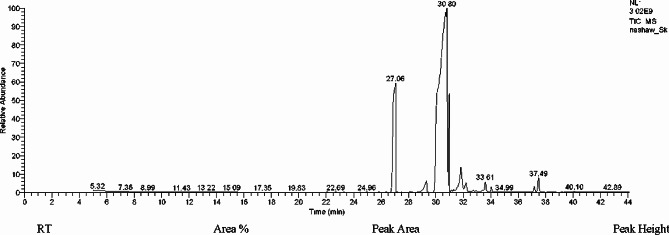
GC-Mass analysis for methanol extract of *S. platensis*

*S. platensis* produces a diverse range of bioactive molecules, making them a rich source of different types of medicines, [39], GC-MS analysis of the methanol extract of *S. platensis* revealed the presence of a group of bioactive compounds as Hexadecanoic acid methyl ester, n-hexadecanoic acid, 9,12-octadecadienoic acid (Z,Z)-, Methyl ester, cis-13-octadecenoic acid, methyl ester, methyl stearate, 12-octadecadienoic acid (Z,Z), Octadecanoic acid, cis-13-eicosenoic acid, Eicosanoic acid, methyl ester, docosanoic acid, methyl ester and Di-isooctyl phthalate (Table 2; Fig. 2), similar findings were obtained by Deyab et al. [40] who reported that the major bioactive components of *S. platensis* methanol extract were Hexadecanoic acid (29%) and 9,12-octadecatrienoic acid methyl ester (24.36%), Awadalla et al. [41] reported the antibacterial effect of the methyl alcohol extract of *S. platensis* against *S. aureus*, *E. coli*, *P. aeruginosa*, *S. typhi* and *K. pneumoniae*. Algal extracts were discovered to have a significant role in the biological synthesis of nanoparticles and the conversion of various forms of heavy metals to their innocuous counterparts [42]. Yuliani et al. [43] dedicated that *S. platensis* methanol extract comprises a variety of fatty acids that could efficiently act as reducing agents for the synthesis of nanoparticles. In the present study, ZnO NPs were biofabricated by the methanol extract of *S. platensis* using zinc acetate dehydrate as a precursor salt.

**Table 2:** Various identified compounds in the methanol extract of *S. platensis*

No	Retention Time (min)	Peak Area %	Compound Name	Molecular Formula	Molecular Weight
1	27.05	25.60	Hexadecenoic acid methyl ester	C_17_H_34_O_2_	270
2	29.31	1.27	n-Hexadecenoic acid	C_16_H_32_O_2_	256
3	30.03	6.26	9,12-Octadecadienoic acid (Z,Z)- methyl ester	C_19_H_34_O_2_	294
4	30.80	52.29	cis-13-Octadecenoic acid, methyl ester	C_19_H_36_O_2_	296
5	30.97	7.91	Methyl stearate	C_19_H_38_O_2_	298
6	31.83	2.31	9,12-Octadecadienoic acid (Z,Z)-	C_18_H_34_O_2_	282
7	32.20	0.85	Octadecanoic acid	C_18_H_36_O_2_	284
8	33.61	0.94	cis-11-Eicosenoic acid	C_20_H_38_O_2_	310
9	34.04	0.47	Eicosanoic acid, methyl ester	C_21_H_42_O	326
10	37.17	0.60	Docosanoic acid, methyl ester	C_23_H_46_O_2_	354
11	37.48	1.51	Di-isooctyl phthalate	C_24_H_38_O_4_	390

### Green synthesis and characterization of ZnO NPs

Zinc acetate dehydrate solution was colorless, after the addition of *S. platensis* methanol extract, the reaction mixture turned green, after 4 h the color of the mixture changed to pale white indicating nanoparticles configuration, the biogenic synthesis of ZnO NPs may be proceeded by one of two mechanisms, the first suggested that Zinc ions (Zn^2+^) were chelated by the biomolecules in the *S. platensis* methanol extract to create complexes, which were then calcined to produce ZnO NPs [44, 45], on the other hand, the second mechanism proposed that *S. platensis* methanol extract compounds reduced Zn^2+^ to Zn metal which reacted with the oxygen dissolved in the solution forming ZnO, moreover, the extract constituents acted as nanoparticle stabilizers that prevented their agglomeration [35].

**Fig. 3:**
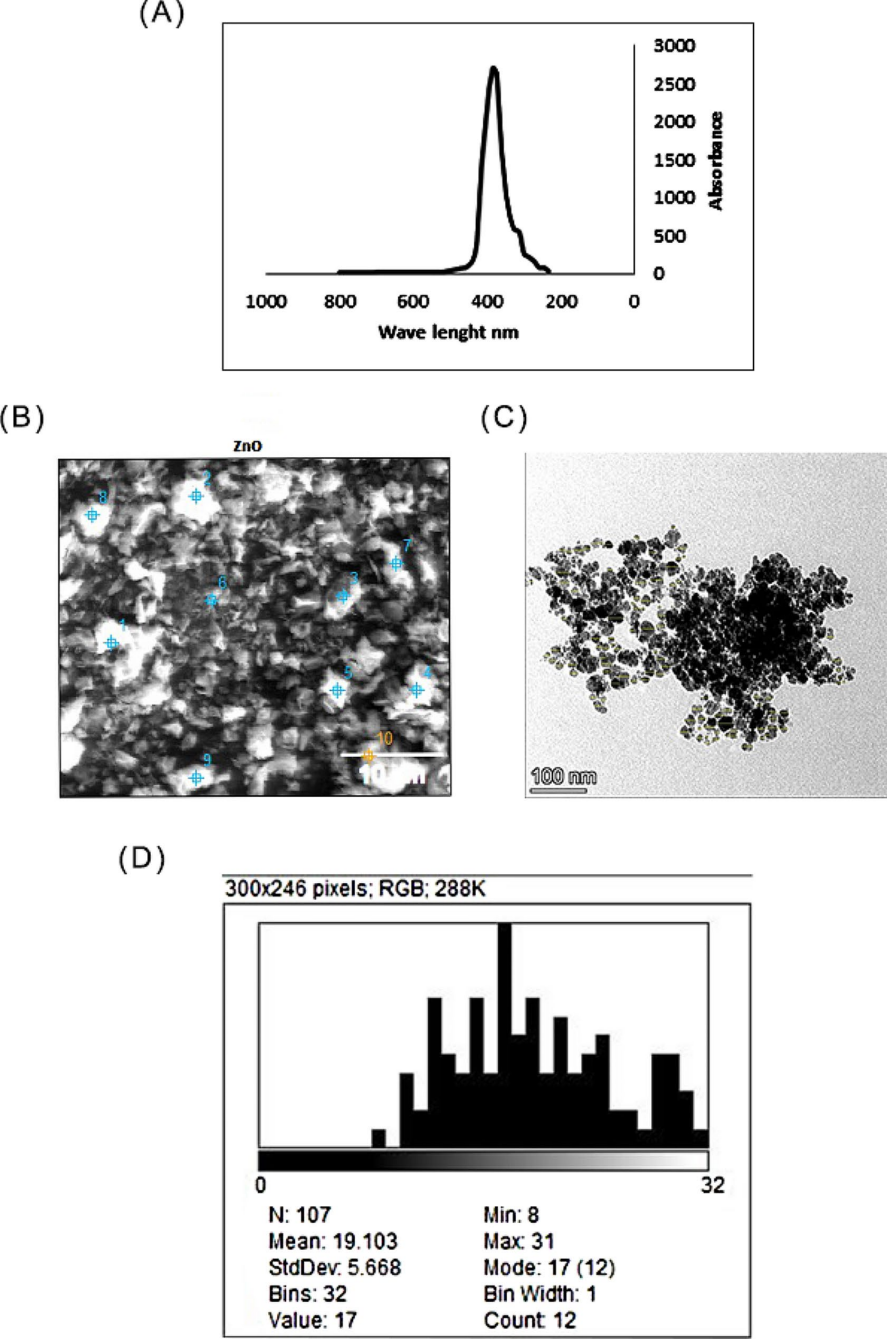
(A) UV-Vis spectra of the green synthesized ZnO NPs; (B) SEM micrograph of the green synthesized ZnO NPs;(C) TEM micrograph of the green synthesized ZnO NPs ;(D) Particle size distribution histogram

The synthesized nanoparticles were characterized before application to identify their chemical and physical properties including their stability, distribution, and composition in addition to their shape and size, green production of ZnO NPs via *S. platensis* methanol extract was confirmed by the UV-visible spectra, which showed the greatest absorbance peak at 372 nm **(**Fig. 3A), the obtained results satisfied the standard ZnO absorption pattern because all metal oxides have wide band gaps and shorter wavelengths [46], this concept supports the obtained results for ZnO NPs. FE-SEM is used to examine the surface morphology of the green synthesized ZnO NPs, also it provides details on composition, crystallography, topology, and surface morphology. The FE-SEM image showed that the ZnO NPs had excellent dispersion and were hexagonal in shape with some rough aggregations (Fig. 3B) which is in agreement with the previous literature [47]. TEM micrograph confirmed the morphology of the particles (Fig. 3C), based on the TEM image the particle sizes were determined by using ImageJ v1.54 g software they were found to be within the range of 8 to 31 nm ( Fig. 3D). The mean particle size was 19.103 ± 5.66 nm, our results are in harmony with the previous reports confirming the antibacterial effectiveness of the biogenic hexagonal-shaped nanoparticles due to their potent penetration ability of the cell wall of the pathogenic bacteria [48], however, the smaller size of particles reflects the effectiveness of the bioactive compounds involved in the green synthesis method via *S. platensis* methanol extract.

**Fig. 4:**
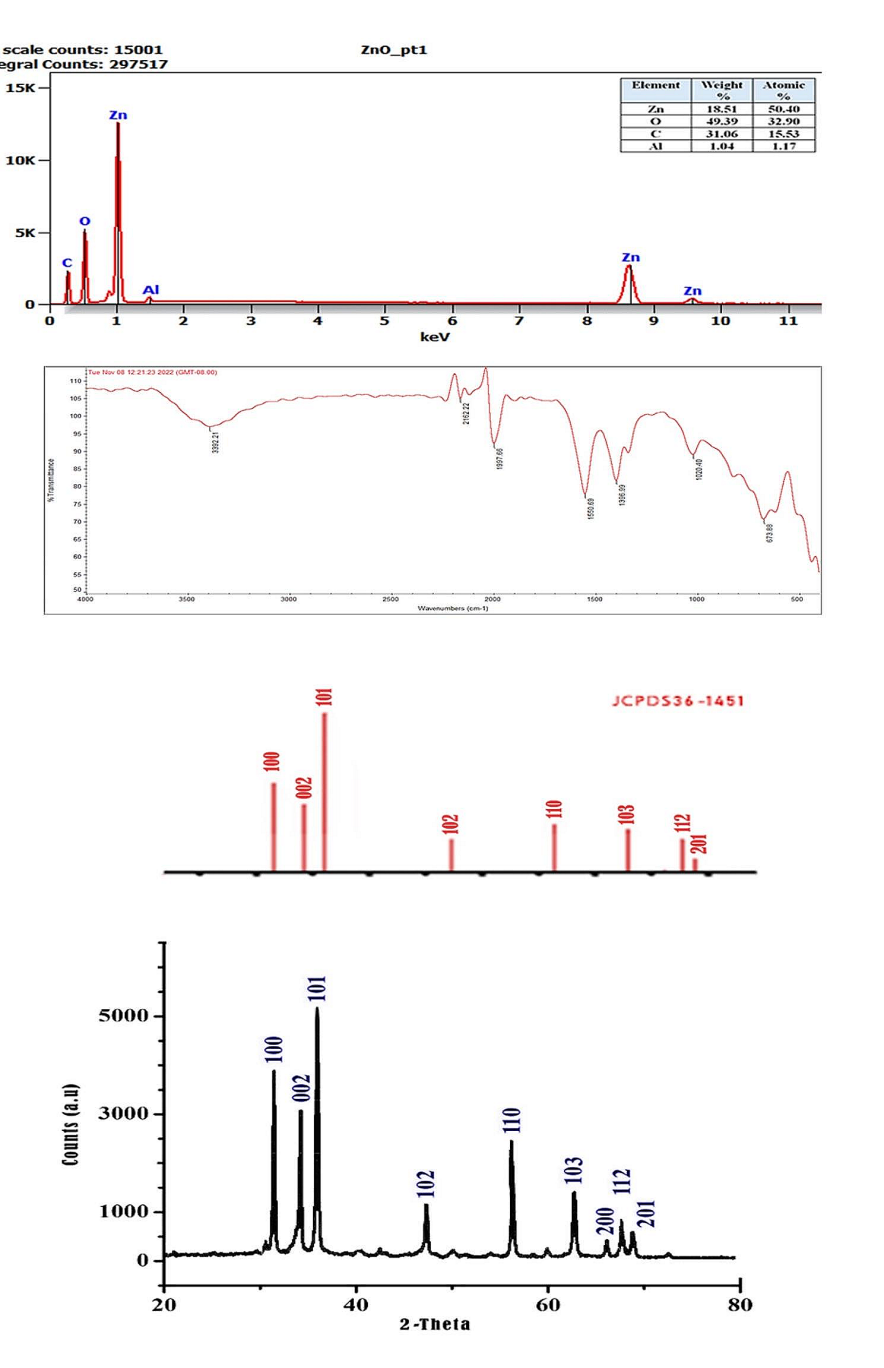
(A) EDX Spectrum of the green synthesized ZnO NPs, (B) FTIR spectrum of the green synthesized ZnO NPs, and (C) XRD pattern of the green synthesized ZnO NPs

The XRD pattern of green ZnO NPs was studied by using Origin 8.5 software. Figure 4A showed sharp diffraction peaks existed at 2θ angles of 31.85°, 34.55°, 36.35°, 47.69°, 56.75°, 63.09°, 66.56°, 68.17° and 69.29° which were corresponded to the crystallographic plans of )100), (002), (101), (102), (110), (103), (200), (112), and (201), these peaks were matching with those of JCPDS card No:36-1451, which confirmed the hexagonal wurtzite structure of ZnO NPs [49], the obtained results are in agreement with those of Faisal et al. [50], the avarage crystallite size of ZnO NPs was calculated by the Debye − Scherrer formula where D = K λ/β Cosθ where D is crystal size, *λ* is the wavelength of the X-ray radiation (*λ* = 0.15406 nm), K is shape factor typically taken as 0.89, β is the full width at high maximum (FWHM) corresponding to 101 planes located at position 36.35º, and θ is the Bragg’s diffraction angle, the mean crystalline size of green ZnO NPs was calculated to be 18.4 nm. The FTIR method was employed to look for potential fragments in the biosynthesized ZnO NPs (Fig. 4B), seven intense peaks were identified, the broad stretch peak at 3392 cm^− 1^ denotes the presence of an O-H stretch band, the width of this peak was attributed to the intra-and inter-molecular hydrogen bonding [51], O = C = O (stretching vibration) was identified as the cause of the absorption peak at 2162 cm^− 1^, the intensity peak at 1997 cm^− 1^ was associated with the C-H stretching of symmetric and asymmetric carbohydrates or lipids [52]. The stretching C = C vibration of the aromatic ring system was shown by the peak seen at 1550 cm^− 1^, the C-N stretching vibration of amino acids was shown by the absorption peak at 1396 cm^− 1^ [51], the C-O stretching bond of the aromatic rings is responsible for the peak at 1020 cm^− 1^ which could potentially be attributed to phenols. The absorption band at 673 cm^− 1^ proved that ZnO NPs were formed successfully. In agreement with our findings it was reported that metals’ oxides were distinguished from absorption bands below 1000 cm^− 1^, the Zn-O stretching band was found around 400–700 cm^− 1^ [53]. The stoichiometry of ZnO NPs was investigated using EDX analysis, the major peak confirms that Zn (50.4%) is the major constituent of the nanostructure, additional peaks of O (32.9%) and C (15.53%) are fundamental elements included in the bioactive constituents of the algal extract, their values may vary depending on several factors such as growth conditions, cultivation methods, and environmental factors Alsaggaf et al. [28], however, 1.17% of Al represented the presence of trace amount of impurity (Fig. 4C).

### Antibacterial vulnerability

**Fig. 5:**
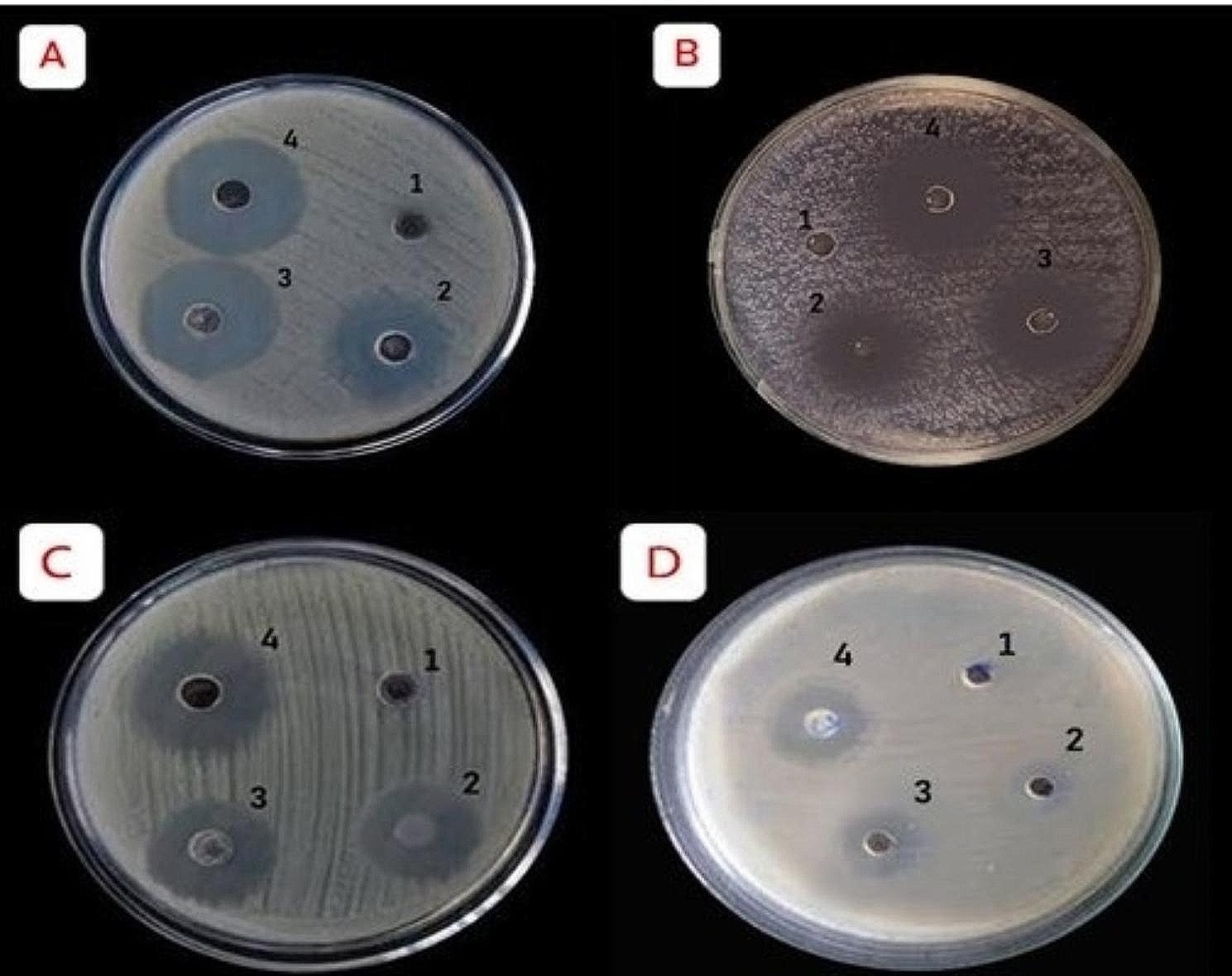
Antibacterial activity of different concentrations of green synthesized ZnO NPs versus *S. aureus* (A), *K. pnuemoniae* (B), *S.pyogenes* (C) and *S. typhi* (D); Negative control (1), ZnO NPs at 10 µg/mL (2), ZnO NPs at 20 µg/mL (3), Tetracyclin (4)

Biogenic ZnO NPs have been considered as an advancement step of next-generation nano-antibiotics used to combat multidrug-resistant pathogens [54], its immense antimicrobial potential versus several pathogenic bacteria was derived from their extraordinary physicochemical features including crystallinity, porosity, particle size, and shape [55]. The antibacterial potentiality of the biogenic ZnO NPs was assessed by measuring the inhibition zone diameter (IZ), the obtained outcomes confirmed that they exhibited excellent antibacterial dose-dependent manner against all challenging bacterial strains (Table 3; Fig. 5), the biosynthesized ZnO NPs demonstrated greater antibacterial efficiency against Gram-positive bacteria compared to Gram-negative ones, this may be due to the composition and structure of the cell wall of Gram-positive bacteria that may enhance the nanoparticles’ ability to adhere to the cell wall, on the other hand Gram-negative bacteria lackes this ability, our outcomes were in line with those of Vijayakumar et al. [56], who stated that biosynthesized ZnO NPs using *L. nobilis* leaf extract had similar antibacterial trend, also the biosynthesized ZnO-NPs using *Mentha mozaffarianii* extract showed significant bioefficiency against *K. pneumoniae* strain with inhibition zones of 14 and 17 mm at ZnO-NP concentrations of 25 and 50 µg/mL respectively [57]. The highest significant activities were recorded for *S.aureus* (Gram-positive representative) and *K. pneumoniae* (Gram-negative representative) at both concentrations (10 and 20 µg/mL), the extra surface area of ZnO NPs that interacts with the bacterial cellular membranes may be the cause of this antibacterial behavior. This interaction may increase the entry of the nanoparticles inside the cells, so compromising their viability [11].

**Table 3:** Inhibition zone diameters of methanol extract of *S. platensis* and green synthesized ZnO NPs versus tested

Microorganisms	Inhibition Zone diameters (mm)		
	Green synthesized ZnO NPs		Tetracycline
	10 µg/mL	20 µg/mL	20 µg/mL
**Gram-Positive Bacteria**			
***S. aureus***	15.47 ± 0.25Ab	17.33 ± 0.06Aa	17.63 ± 0.12Aa
***S. pyogenes***	13.53 ± 0.35Bc	14.57 ± 0.06Bb	15.23 ± 0.06Ba
**Gram-Negative Bacteria**			
***S. typhi***	10.47 ± 0.06Dc	12.57 ± 0.06Cb	13.23 ± 0.15Ca
***K. pneumoniae***	11.43 ± 0.12Cc	14.57 ± 0.06Bb	15.07 ± 0.06Ba

Different small letters indicate significant differences at (*p* < 0.05) among means in the same row and different capital letters indicate significant differences at (*p* < 0.05) among means in the same column. Using: One-way Analysis of Variance test was performed for Mean ± SD & Multiple comparisons between groups through Post Hoc test: Tukey’s test.

### Estimation of MIC and MBC

**Fig. 6:**
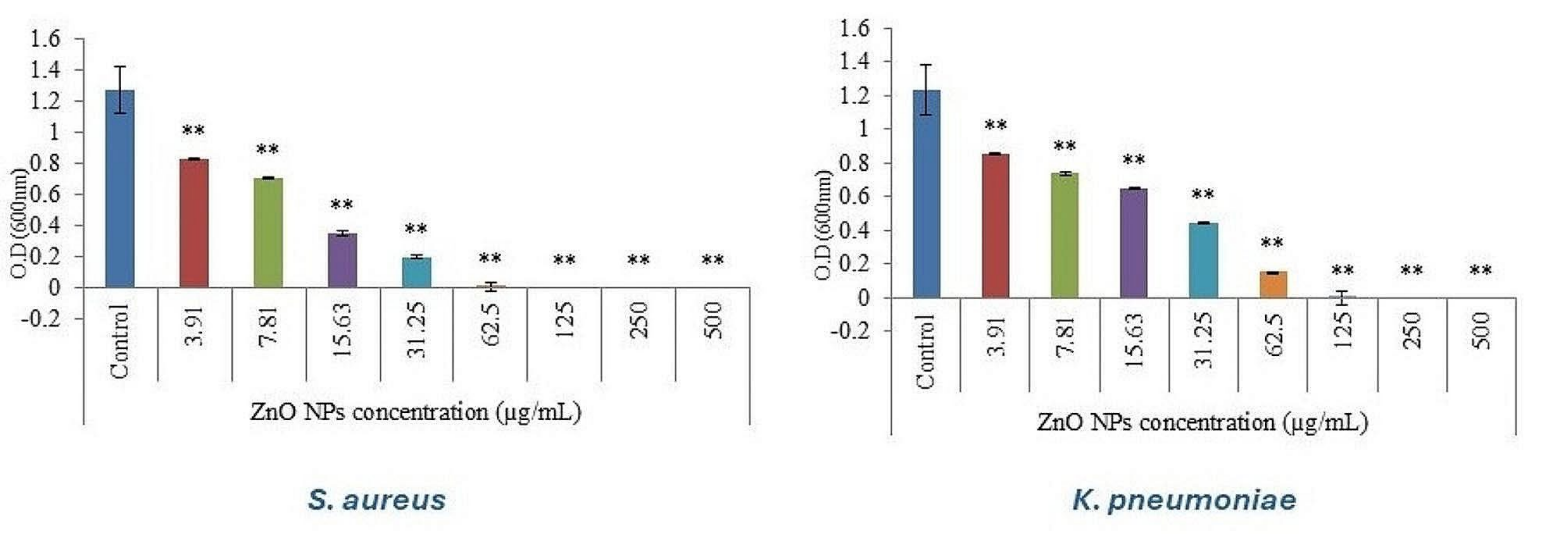
Effect of varying concentrations (µg/ mL) of the green synthesized ZnO NPs on *S. aureus* and *K. pneumoniae*, results are expressed as mean ± SD after triplicate studies. The * and ** in the bars represent S.E. (* *p* < 0.05, ** *p* < 0.01)

After challenging the most sensitive bacterial pathogens with a serial of descending concentrations from 500 to 7.8122 µg/mL of the green synthesized ZnO NPs, both the minimum inhibitory concentration (MIC) and the minimum bactericidal concentration (MBC) were recorded for each pathogen, from the represented results (Fig. 6), it was observed that there is a remarkable directly proportional relation between the reduction in the visible growth of the tested bacterial strains (P˂0.05) versus the gradual increase in the concentration of ZnO NPs, the recorded MIC was 31.25 µg/mL and 62.5 µg/mL for *S. aureus* and *K. pnuemoniae* respectively. Consequently, the minimum bactericidal concentration (MBC) was estimated by plating inoculums from MIC wells onto nutrient agar plates and incubating them at 37 ºC for 24 h, the concentration displaying no visible bacterial growth being recorded as the MBC [58]. The MBCs for *S. aureus* and *K. pnuemoniae* were 125 µg/mL and 250 µg/mL respectively, there has been speculation that the lower susceptibility of Gram-negative bacteria could be related to the more complex cell wall with additional lipopolysaccharides that act as a barrier against antimicrobial agents, also ZnO NPs may have different affinities for the cell surfaces of *S. aureus* and *K. pneumoniae*, leading to variations in their antimicrobial activity, our findings were in line with the previous studies on ZnO NPs mentioned by Yao et al. [59], Mahdavi et al. [60] and Alam et al. [61].

### Estimation of morphological distortion of the bacterial cells using transmission electron microscope examination (TEM)

**Fig. 7:**
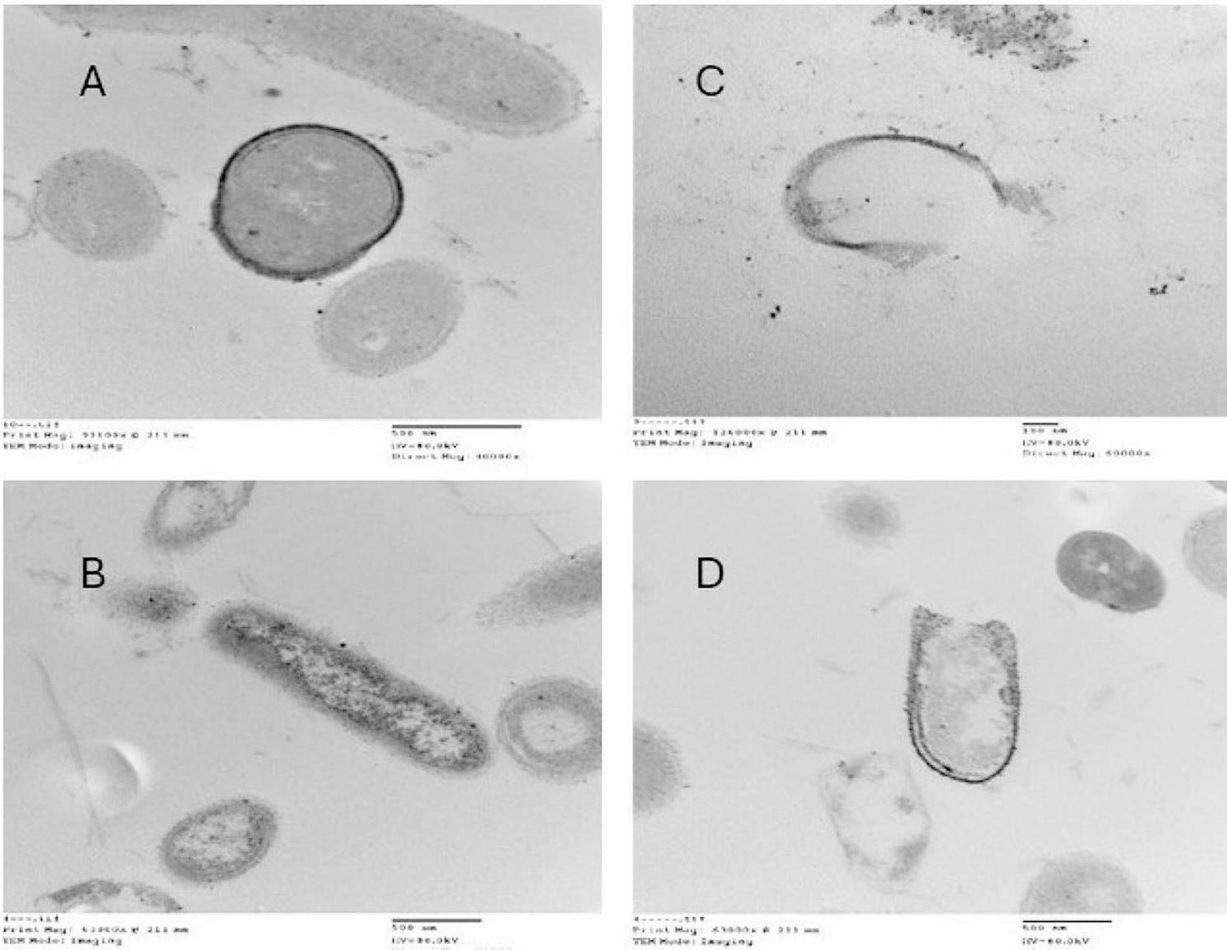
TEM examination (Magnification, 2500 X) of *Staphylococcus aureus* (A,C) and *Klebsiella pneumoniae* (B,D)Where: A and B are the untreated bacterial cells, C and D are the treated bacterial cells by green ZnO NPs

Our findings concerning ultrastructure examination using transmission electron microscopy (TEM) for the two most affected bacterial strains *S. aureus* and *K. pneumonia* (Fig. 7) revealed that treatment with green biosynthesized ZnO NPs first causes damage to the cell membrane before moving to the cytoplasm where they interact with other cell structures, these were agreed with those of El-Deeb et al. [62] and Yagoub et al. [63]. ZnO NPs are hence multi-target antimicribial that affect several bacterial cell structures. However, their primary mode of action is in the cytoplasmic membrane, with other structure effects occurring as a secondary effect after membrane rupture [64].

### Antibiofilm quantification

**Fig. 8:**
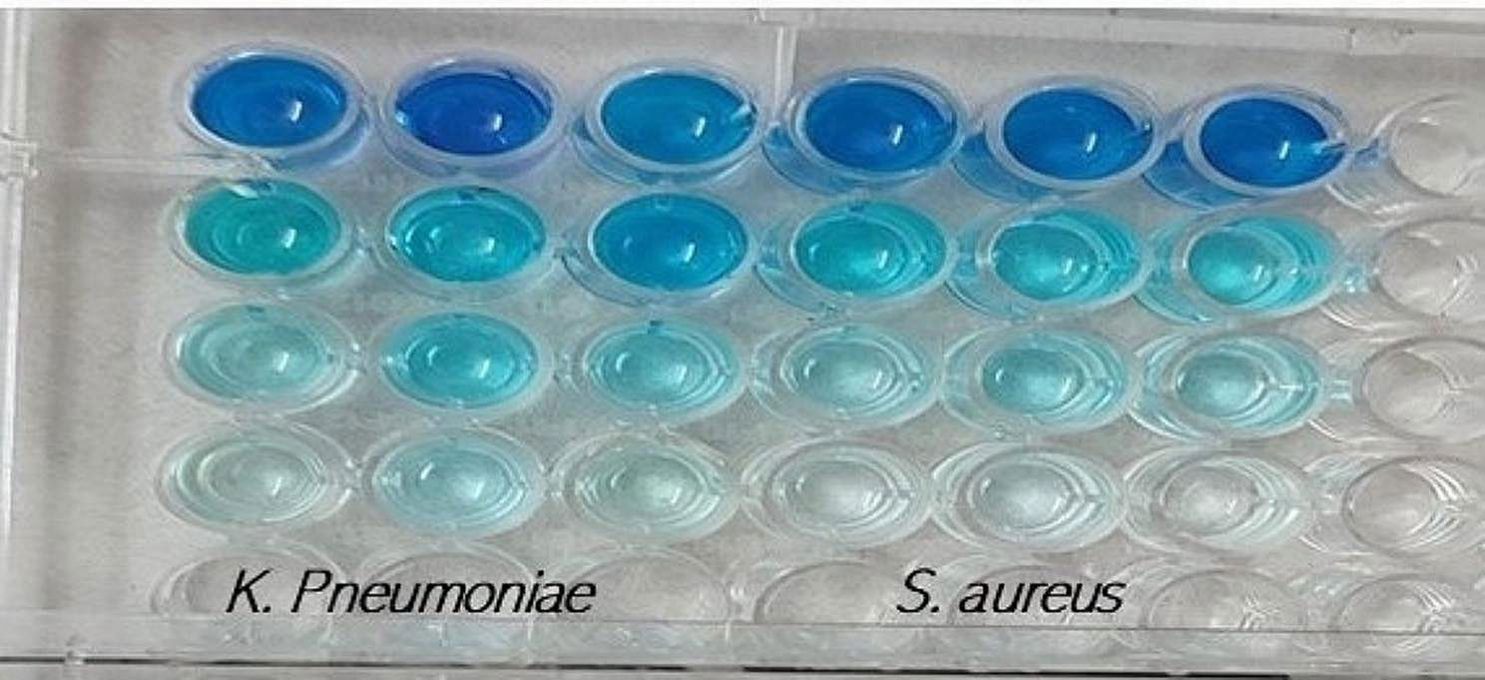
Results of biofilm formation assay via microplate dilution method

Biofilms’ production is a major concern in both the industrial and medicinal fields. Biofilm can cause biofouling in industrial equipment and lead to medical device-associated infections. Clinically, biofilms play an important role in chronic and persistent infections by increasing the antibacterial resistance as well as reducing the immune response [65]. The search for innovative antibiofilm agents could lead to new approaches for controlling infections and provide solutions to biofilm-related problems [66]. According to earlier studies, nanoparticles may have an impact on the life cycles of bacteria, which in turn can alter their activities such as cell-cell communication [65], many biogenic nanoparticles have been shown to successfully eradicate bacterial biofilms [26]. In the present study we investigate the antibiofilm potentiality of ZnO NPs on the challenging biofilm-forming bacterial strains, from the results represented in Fig. 8; Table 4 it was observed that the green ZnO NPs were able to eradicate the biofilm formed by *S. aureus* and *K. Pneumonia* at sub-MICs tested with maximum significant reduction value of 96.7% and 94.8% for the two isolates respectively at a concentration equivalent to 2 MIC, near results were presented by Husain et al. [67] who reported that the biogenic ZnO NPs were able to degrade the biofilms formed by each of *E. coli, S. aureus*, and *P. aeruginosa.* The antibiofilm activity of biogenic ZnO NPs can be explained as they can affect exopolysaccharide synthesis [68], also they can penetrate the biofilm matrix due to their small size and high surface area. Once inside, they can interfere with biofilm architecture by disrupting the extracellular polymeric substances (EPS) that strengthen the biofilm. This disruption weakens the structural integrity of the biofilm, making it more susceptible to further degradation or removal [69].

**Table 4:** Anti-biofilm activity of the green synthesized ZnO NPs against *K. pneumoniae* and *S. aureus*

Concentration of Biogenic ZnO NPs	K. pneumoniae			S. aureus		
	OD	SD	Antibiofilm inhibition percentage (%)	OD	SD	Antibiofilm Inhibition percentage (%)
Blank	0.005	0.002	-	0.005	0.002	-
Control	1.719	0.008	-	1.719	0.008	-
2 MIC	0.095	0.007	94.8	0.080	0.006	96.7
1 MIC	0.193	0.007	89.1	0.168	0.006	91.5
0.5 MIC	0.367	0.002	78.9	0.243	0.003	85.7

OD; optical density, SD; standard deviation

### Biological evaluation of ZnO NPs

#### Antioxidant activity

**Fig. 9:**
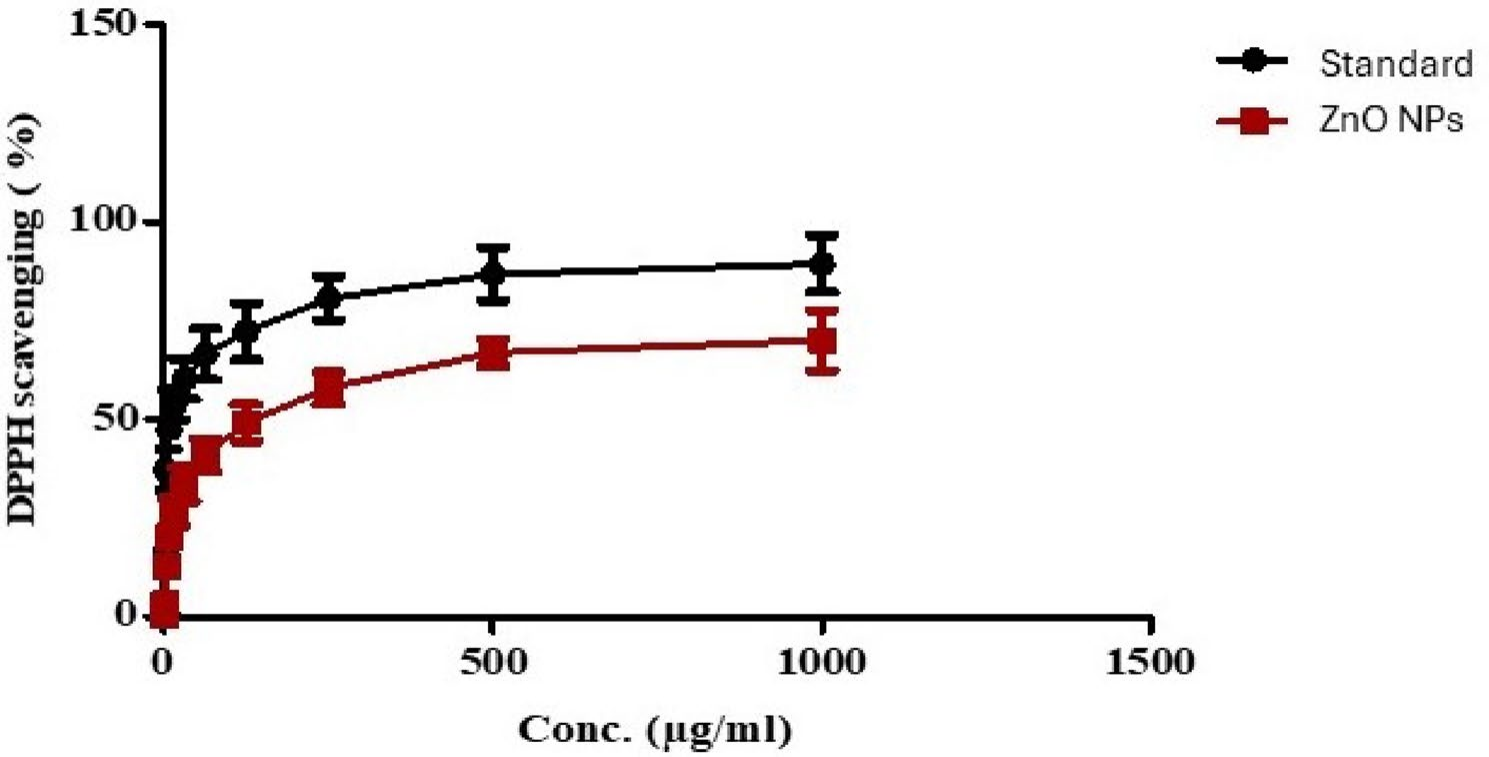
Antioxidant activity of green synthesized ZnO NPs (Data are expressed as means ± S.D) where IC_50_ = 78.35 µg/mL for ZnO NPs, while for standard IC_50_ = 8.1 µg/mL

The DPPH assay is frequently used to evaluate a compound’s antioxidant capacity by determining its ability to scavenge DPPH radicals. This assay indicates the ability of an antioxidant to neutralize or reduce free radicals in living cells [70]. The results illustrated in Fig. (9) revealed a dose-dependent increasing antioxidant activity of the biogenic ZnO NPs, the DPPH assay demonstrated significant radical scavenging activity of ZnO NPs having an IC_50_ value of 78.35 µg/mL relative to ascorbic acid of IC_50_ value 8.1 µg/mL.

Numerous studies have attempted to invent different, useful, and reasonably priced antioxidants with reduced toxicity [71]. In the present investigation, ZnO NPs had an antioxidant role which could be produced due to the presence of hydroxyl groups in metal nanoparticles as illustrated by other research groups [72].

#### Anti-inflammatory activity

**Fig. 10:**
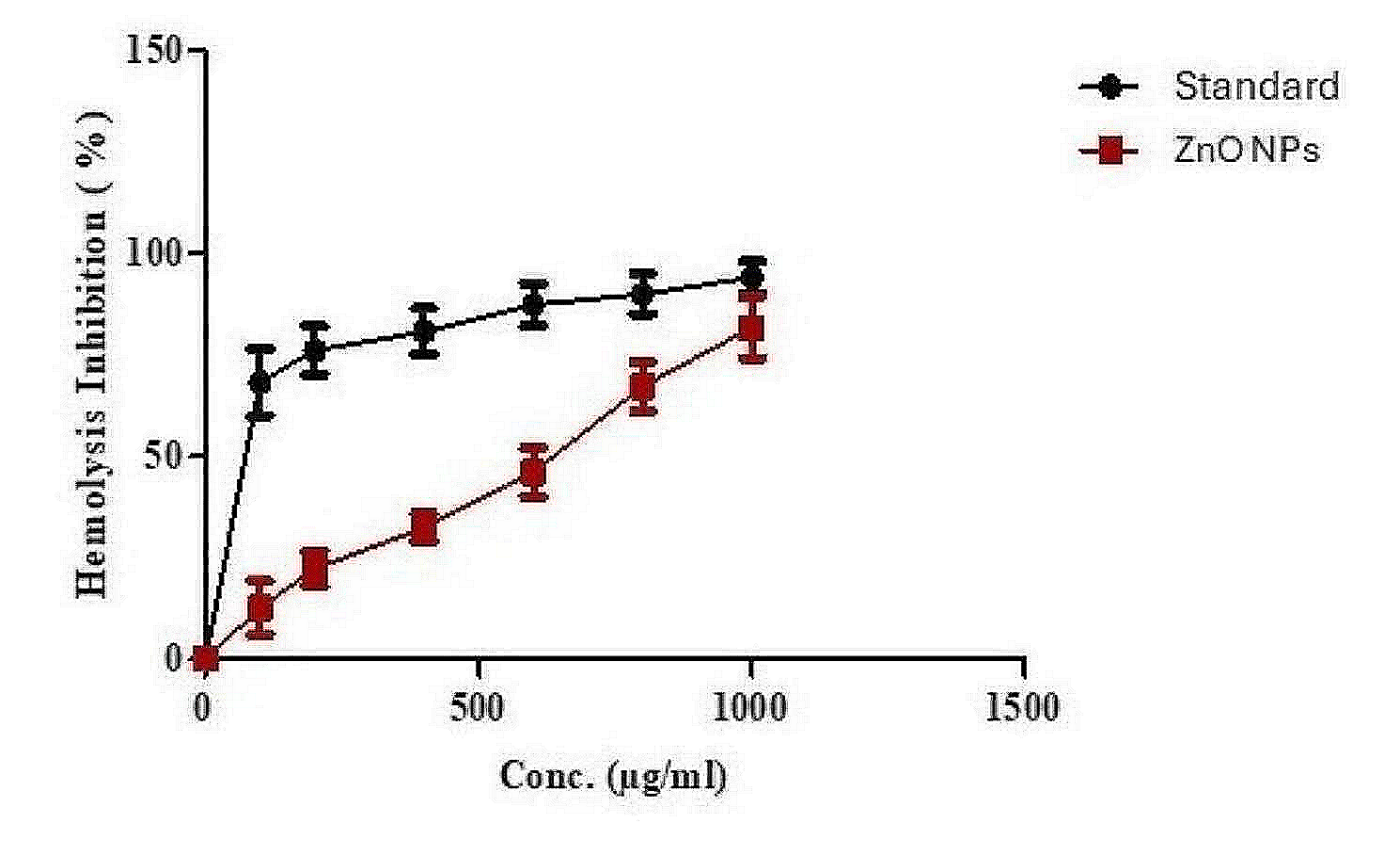
Anti-inflammatory activity of green synthesized ZnO NPs (Data are expressed as means ± S.D)

The membranes of human red blood cells (HRBCs) lysed in the presence of toxic chemicals such as hypotonic solutions, methyl salicylate, or phenylhydrazine, which causes hemolysis and hemoglobin degradation. HRBCs are reported to resemble lysosomes, which lyse and produce enzymes that cause inflammatory responses [73]. Using this technique, green ZnO NPs’ anti-inflammatory activity was measured. The results (Table 5; Fig. 10) revealed the auspicious anti-inflammatory action of green ZnO NPs, where the level of protection elevated upon increasing its concertation as at 1000 µg/ml ZnO NPs significantly (0.005 ≤ P) produced 89.3% hemolysis inhibition. ZnO NPs’ anti-inflammatory effect might be by stabilizing the HRBCs’ membranes via blocking the production of active inflammatory processes and lytic enzymes, or by inhibiting the biomolecules necessary for the creation of the biochemical inflammatory mediators [74].

**Table 5:** Effect of the green synthesized ZnO NPs on hypotonicity induced hemolysis of human red blood cells

Sample	Concentration ug/mL	Mean absorbance ± S.D		Hemolysis Inhibition %
		Hypotonic solution	Isotonic solution	
Control (Indom ethacin)		1.064 ± 0.005	0.001 ± 0.00	
ZnO NPs	1000	0.525 ± 0.015	0.411 ± 0.005	89.3
	800	0.628 ± 0.003	0.344 ± 0.001	73.3
	600	0.800 ± 0.012	0.289 ± 0.004	52.0
	400	0.883 ± 0.002	0.201 ± 0.003	35.9
	200	0.915 ± 0.004	0.134 ± 0.001	26.6
	100	0.939 ± 0.014	0.08 ± 0.005	19.2

Data is expressed as means ± standard deviation

#### Anticoagulation activity

Cardiovascular illnesses are becoming the main source of death and disability globally because of lifestyle changes. Atherosclerosis is the underlying illness, which takes years to manifest and is typically far along before it is discovered. Risk factor adjustment lowers mortality and morbidity, and anticoagulant medication has been successful in preventing thromboembolic diseases, ZnO NPs could play a role in stimulating the production of plasmin, an enzyme that breaks down fibrin crosslinks and dissolves blood clots [75]. The present results revealed the anticoagulant activity of ZnO NPs (Table 6) which follows that of El-Waseif et al. [76] who reported the anticoagulant role of Nano Cellulose-ZnO-Ag Composite. The effectiveness shown by the biogenic ZnO NPs in this study indicated its useful applications in the clinical field for the prevention of thrombosis and its related disorders.

**Table 6:** In vitro coagulation assay of the green synthesized ZnO NPs

PT (Sec)		
Conc. (ug/mL)	ZnO NPs	Heparin
0	11.7	11.7
25	13.5	93.6
50	17.6	115
75	19.3	139
**PTT (Sec)**		
**Conc. (ug/mL)**	**ZnO NPs**	**Heparin**
0	24	24
25	39	125.9
50	49.8	155.2
75	61.2	174.2

#### Antitumor and cytotoxic activities

**Fig. 11:**
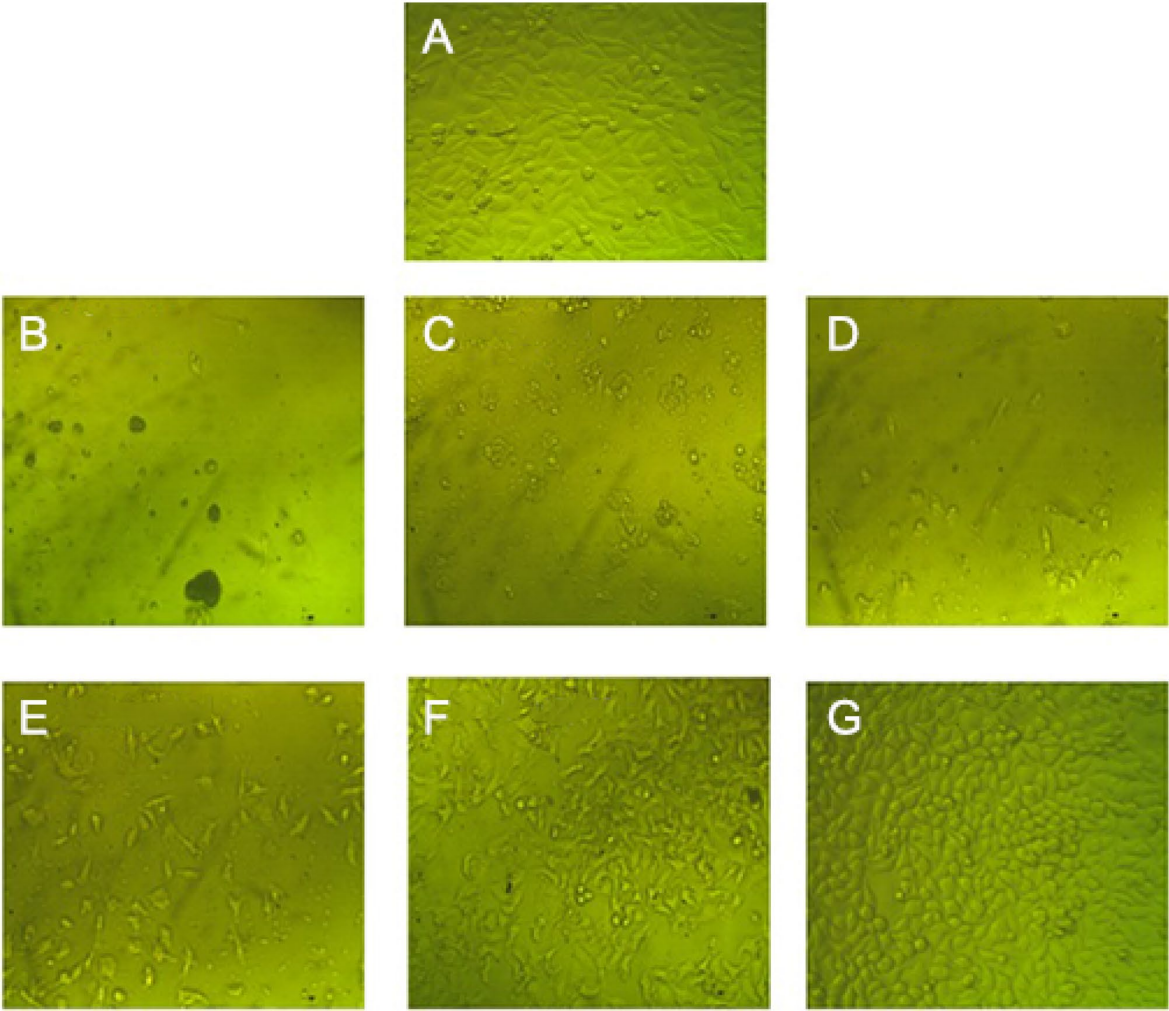
Antitumor activity of green synthesized ZnO NPs on Caco-2 cells (Magnification, 40X); control cells (A), treated cells by green synthesized ZnO NPs at concentrations of 1000 µg/mL (B), 500 µg/mL (C), 250 µg/mL (D), 125 µg/mL (E), 62.5 µg/mL (F), 31.25 µg/mL (G). IC_50_ = 96.25 ± 0.4 µg/mL

Cancer is a free division of malignant cells that can be treated by non-selective traditional methods that cause severe effects on neighboring healthy cells, ZnO NPs were chosen as a promising antitumor nanomaterial considering their features as safety, biocompatibility, and cancer targeting [77], Caco-2 monolayer is a widely used in vitro model of the human small intestine mucosa to predict the absorption efficiency of orally administered drugs, the in vitro Caco-2 cells typically exhibit a monolayer adherent cobblestone-like similar to the lining of the small intestine [78]. In our study green ZnO NPs showed anticancer activity against the Caco-2 cell line with IC_50_ = 96.25 ± 0.4 µg/mL (Table 7; Fig. 11), near findings confirmed the anticancer activity of green ZnO nanoparticles versus HCT-116 and Caco-2 [78].

**Table 7:** In vitro tumor efficacy of the green synthesized ZnO NPs against Caco-2 cell line

Sample	Concentration µg/mL	O.D			Mean O.D	ST.E	Viability %	Toxicity %	IC_50_ µg/mL
Caco-2 Cells		0.547	0.588	0.563	0.566000	0.011930	100	0	
ZnO NPs	1000	0.015	0.016	0.017	0.016000	0.000577	2.826855124	97.17314488	96.25 ± 0.4
	**500**	**0.019**	**0.018**	**0.017**	**0.018000**	**0.000577**	**3.180212014**	**96.81978799**	
	**250**	**0.078**	**0.096**	**0.068**	**0.080667**	**0.008192**	**14.25206125**	**85.74793875**	
	**125**	**0.170**	**0.169**	**0.201**	**0.180000**	**0.010504**	**31.80212014**	**68.19787986**	
	**62.5**	**0.382**	**0.402**	**0.364**	**0.382667**	**0.010975**	**67.60895171**	**32.39104829**	
	**31.25**	**0.546**	**0.580**	**0.572**	**0.566000**	**0.010263**	**100**	**0**	

**Fig. 12:**
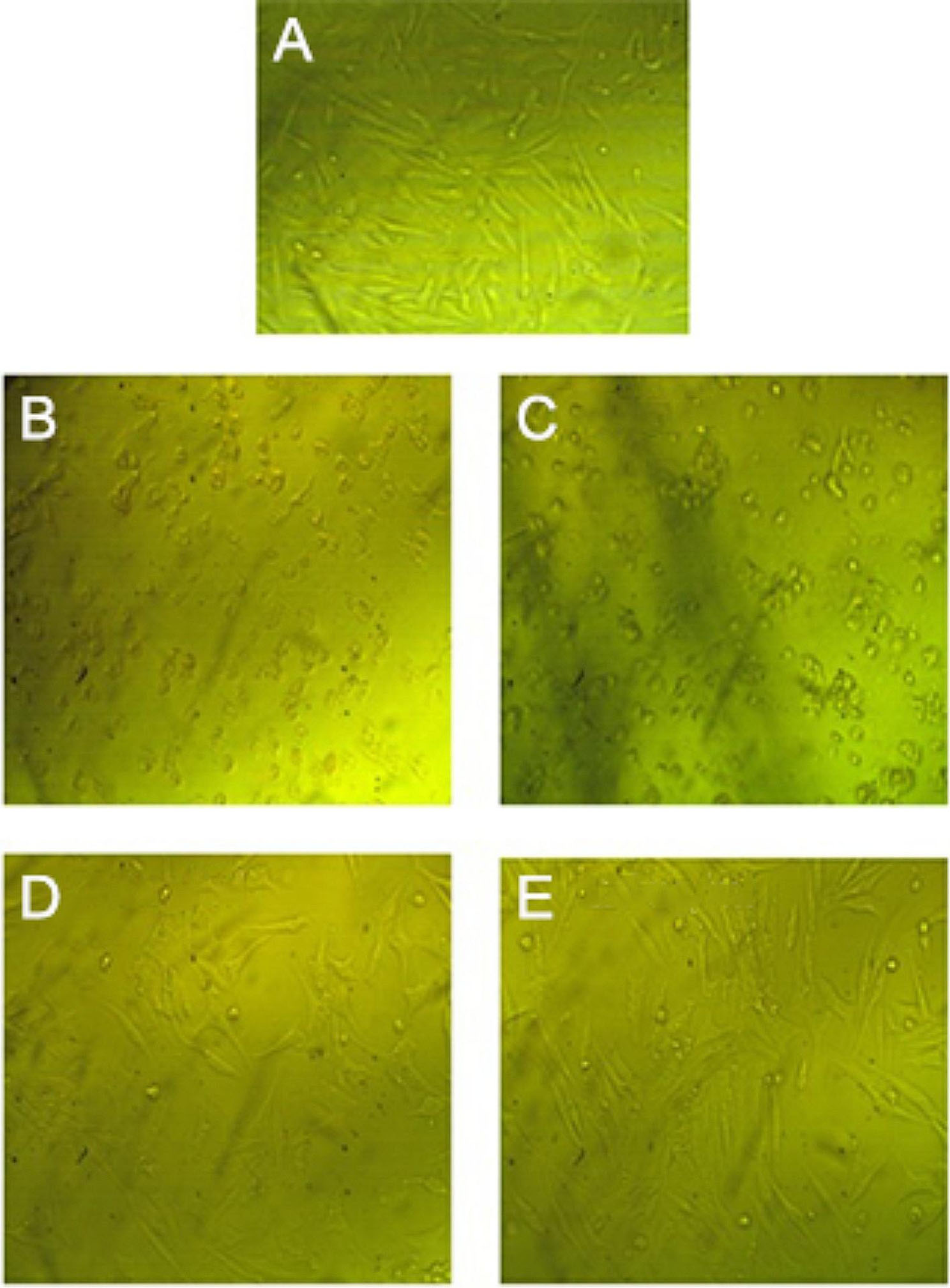
Effect of the green synthesized ZnO NPs on Vero cell line (Magnification, 40X), control cells (A), treated cells by green synthesized ZnO NPs at concentrations of 1000 µg/mL (A), 500 µg/mL (B), 250 µg/mL (C), 125 µg/mL (D), CC_50_ = 301.97 ± 0.7 µg/mL

Since the first stage in determining a synthetic product’s safety on non-cancerous human cells is to evaluate its cytotoxic effects, cell morphology variation was studied to investigate the cytotoxic potentiality of the green synthesized ZnO NPs. The in vitro cytotoxic effect was performed against the Vero cell line and the cell viability was studied using MTT assay, there was a significant dose-dependent decrease in cell viability upon treatment with ZnO NPs (Table 8). Figure 12 shows the change in cell morphology associated with the reduction in viability, the mechanism of formation of rounded cells (indicative of cell death) and leg-like cells (indicative of viable cells) on the treatment of Vero cells by ZnO NPs involves complex cellular responses to nanoparticle-induced stress and subsequent signaling pathway [79], the CC_50_ value was 301.97 ± 0.7 µg/mL, Rao et al. [77] reported that if the CC_50_ value is ≤ 90 g/mL the substance is considered as not cytotoxic, these results revealed that *S.platensis* methanol extract could be used as a reducing agent for ZnO NPs synthesis with powerful antitoxic potential, high selectivity for cancer cells and improved anticancer activity.

**Table 8:** In vitro cytotoxic efficacy of the green synthesized ZnO NPs against Vero cell line

Sample	Concentration µg/mL	O.D			Mean O.D	ST.E	Viability %	Toxicity %	CC_50_ µg/mL
Vero Cells		0.425	0.417	0.421	0.421	0.002309	100	0	
ZnO NPs	1000	0.017	0.016	0.018	0.017	0.000577	4.038004751	95.96199525	301.97 ± 0.7
	**500**	**0.053**	**0.068**	**0.082**	**0.067667**	**0.008373**	**16.07284244**	**83.92715756**	
	**250**	**0.216**	**0.179**	**0.185**	**0.193333**	**0.011465**	**45.92240697**	**54.07759303**	
	**125**	**0.398**	**0.400**	**0.411**	**0.403**	**0.004041**	**95.72446556**	**4.275534442**	
	**62.5**	**0.427**	**0.404**	**0.399**	**0.410**	**0.008622**	**97.3871734**	**2.612826603**	
	**31.25**	**0.419**	**0.425**	**0.406**	**0.416667**	**0.005608**	**98.97070467**	**1.029295329**	

## Conclusion

The main contribution of our manuscript aimed to present a novel, rapid, and sustainable way for large-scale production of green and stable ZnO NPs using *S. platensis* methanol extract, The biosynthesized ZnO NPs showed a characteristic Uv-Vis absorption peak at 372 nm. The XRD pattern also indicated the hexagonal pureWurtzite structure. FE-SEM coupled with EDX, TEM, and FTIR confirmed the formation of NPs with an average size of 19.103 ± 5.66 nm. The biosynthesized nanoparticles manifested potent antimicrobial potentiality versus different significant pathogenic bacterial strains. It was significant that this study also emphasized that green ZnO NPs had pleiotropic biomedical actions including antibiofilm formation, antioxidant, anti-inflammatory, anticoagulant, and antitumor functions. Our findings suggested the possibility of using the methyl alcohol extract of *S. platensis* for synthesizing stable ZnO NPs. so that in the domains of biomedical research, it can be utilized as a steady and safe substitute for synthetic drugs. In the future, more mechanistic, in vitro, and in vivo studies using different animal models are required.

## Data Availability

No datasets were generated or analysed during the current study.
